# Scarcity Enhances Outcome Evaluation in the Present: Electroencephalography Evidence

**DOI:** 10.3390/brainsci12111560

**Published:** 2022-11-17

**Authors:** Liangliang Yi, Daoqun Ding, Xiangyi Zhang, Die Fu

**Affiliations:** 1 Department of Psychology, School of Education Science, Hunan Normal University, Changsha 410081, China; 2 School of Education, Hunan University of Science and Technology, Xiangtan 411201, China; 3 Cognition and Human Behavior Key Laboratory of Hunan Province, Hunan Normal University, Changsha 410081, China; 4 Center for Mind and Brain Science, Hunan Normal University, Changsha 410081, China

**Keywords:** scarcity, evaluation, delay discounting, reward, FRN, frontal asymmetry index

## Abstract

Scarcity goods have generally been perceived as high in value in real-world and empirical studies. However, few studies have investigated this value over time, such as performance in intertemporal decision making. This study’s chief objective was to determine how scarcity evaluation changes temporally. We used the electroencephalogram technique and an outcome evaluation task with the valuation of scarcity and ordinary rewards delivered at different times to explore the effect of scarcity on delay discounting. The feedback-related negativity (FRN) results show that ordinary goods were associated with a more negative amplitude than scarcity goods, and that rewards delivered in the future evoked more negative deflection compared to those delivered immediately. The prominent FRN effect was derived mainly from ordinary trials rather than scarcity trials in the immediate condition and in the future rather than only in the immediate condition. The Frontal Asymmetry Index (FAI) results show that the scarcity condition was associated with greater relative left frontal cortical activity than the ordinary condition when delivered immediately. The frontal asymmetry indicated greater approach motivation. Our electrophysiology data indicate that scarcity goods have a perceived high value, particularly when delivered immediately.

## 1. Introduction

Having too little may change how people look at problems and make decisions [1]. When faced with a lack of, or a lack of access to, goods, services, or resources, such as precious metals, luxury brands, and limited-edition items, perceived scarcity occurs [2,3,4]. Previous psychological research demonstrates that socioeconomic status affects people’s mundane physiology and psychology, which has attracted great attention from a wide range of disciplines [5,6,7,8]. Previous research indicated that poverty or scarcity could impede human cognitive function [9], capturing attention with high resource demand and less susceptibility to other contexts [10]. Poverty could also play an important role in the decision-making area [1,8,11,12,13], such as marketing and purchase intention [4,6,14], sustainable choices [15,16], risk-taking [17], reproductive health [18], and loans [12].

Previous theoretical and empirical research has suggested that people pursue scarce items because they prefer their uniqueness, high value, or specialness [3,4,14,19]. Poverty status, which relates to current and past living situations is also strongly associated with future plans, from a temporal perspective. Evidence from previous studies indicates that scarcity negatively impacts people’s patience for the future, inducing the pursuit of immediate satisfaction rather than long-term profit [8,20]. These myopic decisions could cause behaviors such as failing to do future-oriented planning, engaging in conspicuous consumption, obtaining more loans, and making smaller investments in education and health [1,3,18,21,22].

Previous research has shown that temporal factors are closely associated with decision-making related to poverty or scarcity. For example, a link was found between ecological resource scarcity and pro-environmental actions moderated by future orientation, where a concern for the future (being reminded of water scarcity) promoted water saving [23]. Previous research on intertemporal decisions found steeper temporal discounting with scarcity conditions [24]. Both academic research and marketing practices in real life suggest that product scarcity promotes panic purchase intention [25,26,27]. In addition to lacking substantial items, the scarcity of time, such as limited-time promotion strategies, leads to urgent purchases without careful consideration [28,29,30].

It has been well established that scarcity exerts a strong influence on decision making [22]. However, few studies have considered time discounting of scarcity at different time points. Although an scarcity impairment effect on decision making has been shown in several studies at the theoretically and behavioral levels, the relevant underlying neural mechanisms remain unclear. Recent conceptual and technical advances in electrophysiological techniques have enabled the exploration of those underlying neural mechanisms [31]. Electroencephalogram techniques with convenience and high temporal resolution have been widely used to investigate the temporal neural dynamics of decision making, both from time and frequency domains [32,33,34,35]. With the time domain, previous event-related potential (ERP) studies have showed that feedback-related negativity (FRN) is strongly associated with the valuation process in decision making [35,36,37,38,39,40,41]. FRN, generated by the anterior cingulate cortex with pronounced negative-ongoing deflection distributed over the front-to-central scalp between 200 and 300 ms post-onset of feedback stimulus, reflects the evaluation process in which more negative charges are associated with unexpected or unfavorable events [37,42]. Earlier studies suggested that FRN amplitude is sensitive to the valance of gain and loss, according to the independent coding hypothesis [40,43].

Extensive prior research in the past few years has strongly suggested that FRN serves as an indicator of conflict of evaluation [36,41,44]. In line with the notion that FRN amplitude is only sensitive to the valence of gain and loss but not sensitive to the magnitude of feedback, there have been some inconsistent results that do not support the independent coding hypothesis [38,40,45,46]. For example, a growing body of follow-up research has found that the FRN amplitude does distinguish between scale and magnitude on both positive and negative feedback frames [43,46,47], or the interplay between valance and magnitude [48]. FRN provides useful information in the process of feedback evaluation, so the expectation for a large reward that is not met may induce greater neural activity in the brain, characterized as greater FRN [48,49].

With the frequency domain, accumulating evidence highlights the frontal asymmetry index (FAI) to uncover the potential underlying neural mechanism [33,34,50,51]. Research on the spectrum analysis of brain oscillation has yielded substantial results on the potential mechanisms underlying the brain process [34,50]. For example, alpha band power was found to be inversely related to cortical activity with inhibiting the contralateral frontal region [52,53]. Numerous previous studies focused on the affective valence model of frontal asymmetry, but more recent research strongly emphasized the motivational direction model of frontal asymmetry, in which greater relative left frontal cortical activity was associated with approach motivation [34,50,52,53]. For feedback processing in decision making, the FAI was found to be associated with reinforcement learning, as indicated by FRN [32]. In summary, the impact of scarcity on decision making has been an important issue in the area of neuroeconomics. Nevertheless, little attention has been paid to the evaluation of scarcity information between the present and future, much less the neural cognitive base. The aim of the current study was to assess the effect of scarcity information on evaluation processing, which delivers the reward now or in the future. Here, we investigated this question using the technique of EEG by measuring the electrophysiological markers from FRN and the FAI during the feedback in the evaluation processing of intertemporal choice.

## 2. Materials and Methods

### 2.1. Participants

Forty-four right-handed, healthy undergraduate students (26 females; M = 20.08 years, SD = 2.13 years) participated in this study. All participants had normal or correct-to-normal vision, with no brain injury or neurological or psychiatric disorders. The participants provided written informed consent and received RMB 40 (~USD 6.15).

### 2.2. Stimuli and Procedures

A modification of the previously used outcome evaluation paradigm was administered [54,55,56]. The participants had to make small point or big point choices with selection one of two rectangles between left and right. People received different rewards based on the outcome of their bet. Points one to five meant small and six to ten meant big. There were two types of monetary rewards as feedback, scarcity items, and ordinary items. Ordinary feedback was represented by currency notes (picture from notes with a face value of RMB 10), and scarcity feedback was represented by Olympic commemorative bank notes. Both notes have the same denomination (RMB 10, ~USD 1.54), and a similar design and circulation, but scarcity was assigned to commemorative bank notes owing to their limited quantity (six million). Prior to the task, we presented the issuing information of commemorative bank notes, as well as the delivery time points of now and one month later. There were four conditions for a correct response: scarcity/now, scarcity/future, ordinary/now, and ordinary/future. Each condition included 60 trials.

**Figure 1:**
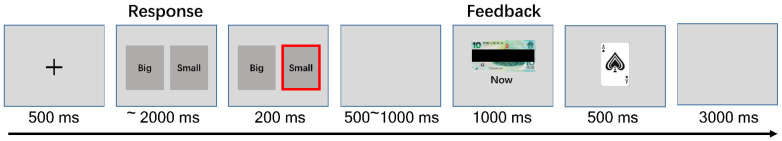
Shows that the participant was required to gamble the two porkers (“Big” or “Small”). The chosen option would be highlighted by a thick red border. All kinds of outcome feedback were presented randomly over the four conditions (now/ordinary; now/scarcity; future/ordinary; future/scarcity). In this example, the participant chose “Small” and gained an Olympic commemorative banknote “now”, then the chosen poker was presented. EEG was only for the phase of feedback. In the formal experiment, the black rectangle on the banknote was not shown in the phase of feedback.

The time course of a trial with Now/Scarcity feedback is illustrated in Figure 1. The experiment was conducted in an electrically shielded room. Each trial started with a fixation cross presented for 500 ms, followed by two rectangles. The gambling choices, small (小) and large (大), were simultaneously presented in one of the two rectangles. The small and large locations of left and right were balanced across the participants. Participants selected one option by pressing the left joystick key with their thumb of left thumb and pressing the right joystick key with their right thumb, within a 2000 ms time limit. The practice trials before the formal task made sure participants performed the pressing task with their thumb, avoiding external muscle movement. The outline of the selected rectangle was thickened red for 200 ms, followed by a blank screen for a random time interval (500 to 1000 ms). Afterward, feedback was presented for 1000 ms, irrespective of participation selection. The feedback consisted of two types of information. First was the win or the null gain feedback information. The win reward feedback was either an Olympic commemorative bank note, or an ordinary currency note, and the null gain feedback had the number 0. (b) The temporal information of the reward would be delivered as follows: “Now“ (今天) or “After one month” (一个月). Then, the poker point, one number from 1 to 10, indicated that the actual point was presented for 500 ms. The next trial began 3000 ms after the feedback offset. The whole feedback was presented in the center of the screen with a width of 2.5° and height of 1.5° (zero for wrong choice without time information with a width of 0.2° and height of 0.5°). The software package E–prime 2.0 (Psychological Software Tools, Pittsburgh, PA, USA) was used for stimulus presentation.

To familiarize themselves with the stimuli, participants performed 15 trials before the formal gambling task. Each condition (now/ordinary, now/scarcity, future/ordinary and future/scarcity) included 60 trials. In order to enhance the authenticity of the task, the whole task contained a larger part of win feedback (240 trials) and a small part of null gain feedback (60 trials). The null gain served as the filling trials, making the task look like the true gamble in real life. EEG analysis only focused on the feedback of the win response. All 300 instances of feedback were presented with random sequences and divided into five blocks each with 60 trials. After each block, participants could take a self-timed rest and were presented with the number of correct choices and how much they had earned in the block. Similarly to prior research, at the beginning of the experiment, participants were informed that they would receive actual monetary rewards based on random selection of one trial of their choice. That is, the participant would receive a monetary reward immediately or one month later, corresponding to a randomly selected option [57,58]. However, similarly to a previous study [58,59], once the experimental had ended, regardless of the randomly trial they selected, participants debriefed as to the study’s true aims, and all the participants actually received RMB 40 immediately after the experiment.

### 2.3. Electrophysiological Recording, Processing and Analysis

The electrophysiological (EEG) activity was recorded from 64 scalp sites using a Quik-Cap with sintered Ag/AgCl electrodes (NeuroScan Inc., Charlotte, NC, USA) according to the extended International 10–20 System. Vertical electrooculogram (EVOG) was recorded above and below the left eye, and horizontal electrooculogram (HEOG) was recorded from two electrodes placed laterally to the left and right eyes. During recording, all electrodes were referenced online to left mastoid, impedances were maintained below 10 kΩ, data were sampled at 500 Hz, and all the data were bandpass-filtered (0.01–100 Hz) online.

EEG data were processed offline with NeuroScan software (Scan 4.5). Raw EEG data were bandpass-filtered (0.01–30 Hz) and re-referenced offline to the average of the right and left mastoids. Ocular artifacts were corrected using a regression eye-movement correction algorithm. EEG epochs of 1000 ms, with 200 ms prior the feedback onset severed as baseline, were extracted from continuous EEGs. Epochs that contained voltage changes exceeding ±80 μV for all scalp sites were excluded.

Based on previous research [40,41,57] and grand average waveforms of the present study, mean amplitude of FRN was measured via the electrodes of Fz, FC3, FCz, FC4, and Cz between 220 and 270 ms post feedback. A 2 (time: immediate and future) × 2 (reward type: scarcity and ordinary) repeated measures ANOVA was conducted on mean amplitude FRN with SPSS 22.0. To compare scarcity condition and ordinary condition directly, the difference FRN(dFRN) was conducted by subtracting waves of the now condition from the future condition. The mean amplitude of the five electrodes that were the same as FRN between 220 ms and 270 ms were measured. Another repeated measure ANOVA with reward type (scarcity and ordinary) was conducted on dFRN.

Apart from inducing the time domain, the frequency domain, such as the frontal asymmetry index (FAI), was administered based on pre-proceed epoch EEG data [33,34,53]. For feedback, the 1000 ms epochs were extracted and submitted to a fast-Fourier transformation using a 50%-overlapping Hamming window. Power spectra were calculated for alpha band activity (8–13 Hz) and were averaged across all epochs on each condition. Similarly to prior studies, the FAI was calculated by natural log-transformed alpha power scores at the right frontal site (F4) minus alpha power at the frontal site (F3) [32,50,60]. A 2 (time: immediate and future) × 2 (reward type: scarcity and ordinary) repeated measures ANOVA was conducted on the FAI. With all ANOVA, degrees of freedom were corrected by Greenhouse–Geisser correction whenever appropriate. The effect sizes were reported as partial eta-squared (part.η^2^).

## 3. Results

**Figure 2:**
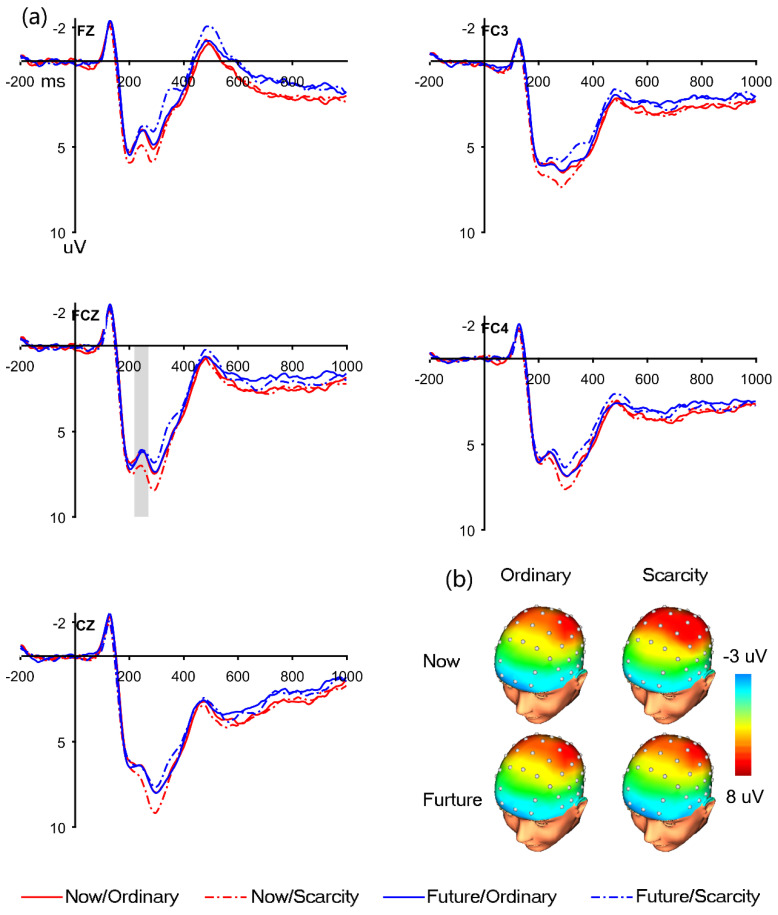
Shows the grand averaged waveforms of FRN. In panel (**a**), electrode points were selected as Fz, FC3, FCz, FC4, and Cz. The solid red line is Now/Ordinary, the dash−dotted red line is Now/Scarcity, the solid blue line is Future/Ordinary, and the dash−dotted blue line is Future/Scarcity. The shadow of the FCZ indicates FRN, and the time point is 220–270 ms. The average amplitude (uV) of 220–270 ms was selected for the EEG topographic map in panel (**b**).

The grand average of FRN and topography maps are displayed in Figure 2. The ANOVA showed significant main effects of reward type (F(1, 43) = 6.781, *p* = 0.013, part.η^2^ = 0.136), with a larger negative deflection elicited for the ordinary items versus the scarcity items (5.738 ± 0.674 μV vs. 6.189 ± 0.675 μV). The main effect of time was significant (F(1, 43) = 4.932, *p* = 0.032, part.η^2^ = 0.103), and a more negative deflection was associated with future than immediate (5.819 ± 0.650 μV vs. 6.108 ± 0.694 μV). Additionally, a significant time × reward type interaction was found (F(1, 43) = 4.376, *p* = 0.042, part.η^2^ = 0.092). The Simple effect analysis showed that ordinary items (5.810 ± 0.659 μV) were associated with larger FRN than scarcity items (6.569 ± 0.721 μV) when delivered immediately (*p* = 0.012), and for scarcity trials, the future condition (6.569 ± 0.721 μV) exhibited a more negative amplitude over the immediate condition (5.674 ± 0.700 μV, *p* = 0.004).

**Figure 3:**
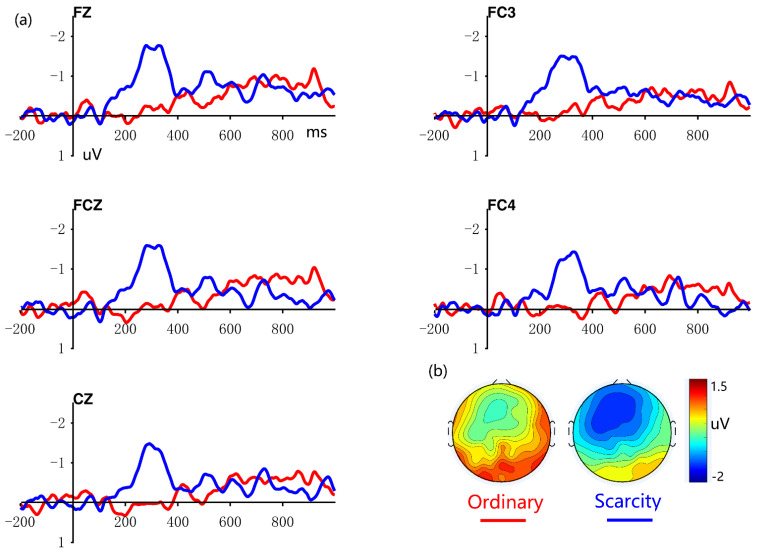
Shows difference waveforms and topographic maps for ordinary and scarcity conditions. The Electrode points Fz, FC3, FCz, FC4, and Cz were selected in panel (**a**). Difference waves were created by subtracting now from future. The red lines represent the ordinary condition, whereas the blue lines represent the scarcity condition. Panel (**b**) indicates the average wave values (uV) during which the dFRN was evaluated (220–270 ms). The left is the ordinary condition, whereas the right is the scarcity condition.

The grand average of dFRN and topography maps of ordinary and scarcity are illustrated in Figure 3. The main effects of the reward type was significant (F(1, 43) = 4.812, *p* = 0.034, part.η^2^ = 0.101), and more negative dFRN was associated with the scarcity condition than the ordinary condition (0.028 ± 2.53 uV vs. −0.926 ± 0.299 uV).

**Figure 4:**
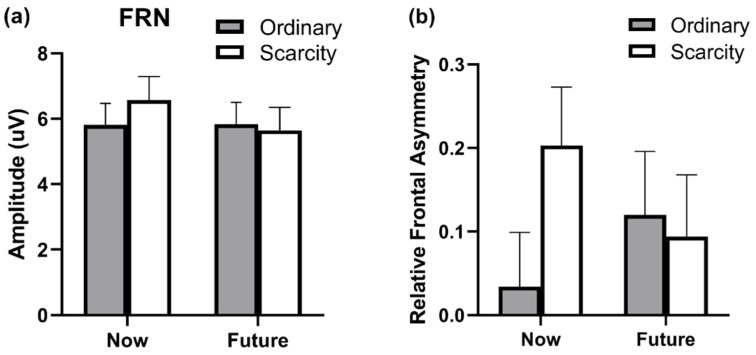
Panel (**a**) shows the mean amplitude values (uV) averaged over five electrodes (Fz, FC3, FCz, FC4, and Cz) across four types of conditions. Panel (**b**) shows frontal alpha asymmetry. The alpha power in the phase of feedback was extracted, and natural log transformation was performed. Then, asymmetry was calculated by subtracting power at the left electrode (F3) from power at the right electrode (F4). Error bars indicate one standard error.

Panel B of Figure 4 depicted the result of the FAI. The ANOVA performed on FAI revealed a significant interaction effect of time and reward type, F(1, 43) = 4.283, *p* = 0.045, part.η^2^ = 0.091. Simple effect analysis showed that for the now condition, the scarcity item was associated with greater relative left frontal cortical activity (0.203 ± 0.070) than the ordinary item (0.034 ± 0.065). There was no significant interaction effect between the ordinary item (0.120 ± 0.076) and the scarcity item (0.094 ± 0.074), *p* = 0.76 for the future condition (*p* = 0.002). There was no significant main effect of reward type (F(1, 43) = 1.920, *p* = 0.173, part.η^2^ = 0.043) and delivered time (F(1, 43) = 0.006, *p* = 0.082, part.η^2^ = 0.001).

## 4. Discussion

We implemented an evaluation task in which the participants had to gamble using a computer and receive different rewards delivered either now or later. The main purpose of this study was to test scarcity goods and ordinary goods with diverse evaluations, especially those goods that were delivered at different times. By measuring FRN and the FAI, we observed more negative deflection for scarcity and immediate conditions, as well as the interplay between the reward item and time for FRN and the FAI.

### 4.1. Scarcity Highlights the Valuation of Items

Scarcity cues often enhance people’s valuation of goods because scarcity is a more perceived value and more prone to be possessed by individuals [2]. The FRN and FAI results of the current experiment confirm this idea. The FRN results of this study show that ordinary items elicited a larger negative deflection than scarcity items. Past research has strongly suggested that FRN serves as an indicator of conflict of evaluation, and FRN amplitude is sensitive to valence as well as magnitude [38,45,46,47], with more negative deflection associated with a negative outcome or outcome worse than expected [36,41,44]. A growing body of literature contends that there is an exception for larger rewards, but if such rewards are not met, they may be associated with greater brain activity, indicated by greater FRN [38,48,49]. The current FRN result supports this notion, as ordinary items elicited pronounced negativity for FRN for scarcity items. In the feedback stimuli of the present experiment, the Olympic commemorative bank notes and ordinary currency notes both could be circulated in the currency market with the same value. Commemorative bank notes with limited issues are scarcity items, which have a higher worth and expected value than ordinary currency notes. In practice, when ordinary currency notes were delivered as feedback stimuli, the result was worse than expected, which is perceived as an unfavorable outcome compared to a high-valued commemorative bank note [47,61]. Evidence from previous decision-making studies suggests that unfavorable feedback decreases dopamine activity but increases ACC activation, accompanied by greater FRN [37,39,46,49], which in turn means a high valuation for scarcity items. This interpretation is supported by a similar evaluation paradigm from Schmidt with four choices: A small magnitude elicited more pronounced negative raw FRN compared to a large magnitude (1 cent vs. 10 cents in German) for the same duration of reward positivity [55].

The result of the FAI further bolsters the highlighting effect of scarcity on the evaluation process. The asymmetry scores obtained in the present results reflect greater relative left frontal cortical activity associated with the scarcity condition than the ordinary condition when delivering the reward now. Evidence from previous psychophysiology studies suggests greater left than right frontal cortical activation, indicating a motivation approach [34,50,60,62]. For the present study, the greater relative left frontal cortical activity associated with the scarcity condition suggests a greater readiness or approach orientation to Olympic commemorative bank notes. The asymmetric activity of present study is in line with that of previous similar studies [50,52,63]. Similar greater relative left frontal cortical activity demonstrated an associated approach motivation, such as apparel product attractiveness [63] and an appetizing dessert [64]. The results of previous neuroimage studies may shed light on neural base asymmetry. It was proposed that the prefrontal cortex plays an important role in the implementation of action, and the activity of the left dorsolateral prefrontal cortex and the inferior frontal gyrus was associated with preparation for goal-directed action [65,66]. The present results together with previous results suggest that scarcity information is associated with greater approach motivation.

There is ample evidence that scarcity goods are perceived as high in value in behavioral investigation. For example, scarce goods were perceived as higher in value compared to ordinary goods in marketing [14,67], and a limited-edition product could provide important information for individuals to classify the commodity scarcity [3]. An early study that manipulated equivalent goods with scarcity and abundant conditions showed that more participants chose scarcity goods and showed a higher willingness to accept the evaluation process [68]. Commodity theory claims that goods are valued to the extent to which they are unavailable [4]. The scarcer the commodity, the greater its uniqueness and the higher its value [4]. Based on previous research and our present results, we argue that scarcity cues may enhance product value [2]. From the individual development perspective, despite cultural differences [11], the pursuit of scarcity may emerge at an very early stage of human development [69,70].

### 4.2. Time Delay Affects Perceived Value

Outcome evaluation was affected by delivery time. Evidence from temporal discounting research has identified the myopic decision that people prefer immediate but small rewards compared to later but larger rewards [71]. The present result of FRN, where a future condition had a larger negative deflection, aligns with that of previous research. A study with a similar paradigm and reward magnitude (10 Yuan in China) showed greater FRN for a delayed reward rather than an immediate reward in the gain condition, which is consistent with the FRN results of this study [42]. Furthermore, taking a step further by subdividing the temporal into now, one week, and one month, the results show greater FRN for the future time conditions [72]. Interestingly, the FRN amplitude increased, even showing a linear trend with time passage, where a reward delivered after one month evoked more negative deflection than that delivered after one week [58].

A part of a previous investigation about evaluation process did not use the identical paradigm as the present study. Some experimental tasks were designed with positive and negative feedback and analyzed with different waves by subtracting the raw FRN responses to the gain condition from the loss condition [56]. Other studies did not even report the statistical results of the raw FRN. Interestingly, the current results coincide with those of various previous studies by intensively scrutinizing the intuitionistic grand average waveforms and the interaction effect, especially the interplay between time delay and valance from related research. For example, in a similar task conducted by Cherniawsky and Holroyd with feedback magnitude × time delay, the grand average FRN waveforms showed the minimal FRN amplitude for large magnitude-immediate trials, which is consistent with our results (Figure 2, with statistical values for reward positivity but not the raw FRN) [54]. In a similar vein, the delayed trials elicited larger FRN deflection than immediate trials with positive feedback in both young and adult groups (panels a and b in Figure 1, with the analysis of difference waves by subtracting FRN elicited gains from the FRN elicited by losses but not the three-way interaction effect of raw FRN) [56]. In a more recent study on the residential mobility mindset on temporal discounting, the grand average waveforms showed greater FRN associated with immediate trials in the gain framework and greater FRN associated with future trials in the gain framework (Figure 2 in study 2, which reported significant temporal main effects but could not find the specific amplitude) [36]. Considering previous experiments and present data, we believe that people have an increased propensity to favor immediate feedback and consider future feedback as unfavorable, resulting in greater FRN.

### 4.3. Temporal Interaction with Scarcity

The significant interaction effect observed in the current research indicates the delivered time and reward type work in an interactive fashion. Less negative FRN was associated with scarce commodities rather than ordinary commodities when delivered immediately in the current study, which suggests that people pursue high-value scarcity items, which aligns with previous research findings [47,61]. Additionally, the propensity for immediate scarcity feedback was further supported by the result of the FAI. Combining smaller FRN and greater relative left frontal cortical activity, we suspect that participants make more exceptions for scarcity items when delivered immediately. Considering the notion that more negative FRN indicates an outcome that is worse than expected, unfavorable, or low-value [47,61], future conditions associated with greater FRN compared to immediate conditions can account for people being inclined to prefer immediate rewards over delayed ones. Studies on intertemporal decision-making yielded consistent results in that future rewards were steeply discounted [36]. This trend is confirmed by the current FRN results associated with scarcity conditions. With the passage of time, future scarcity rewards tended to be more undervalued; this was represented as greater FRN elicited by the future reward as opposed to the immediate reward. The dFRN, distinguishing between the now condition and future condition, provide more information now and in the future [54]. The dFRN of scarcity trials was associated with more negative deflection than ordinary trials. Although there have been few studies about dFRN like the current study (most have focused on win vs. lose) [42,56], the result of present difference waves provides causal evidence to suggest that scarcity information now and in the future is associated with a greater response of the brain.

Together with the results of FRN, dFRN, and the FAI, we posit that there is no direct evidence to show a steep temporal devaluation of ordinary goods. Moreover, there is no significant difference between scarcity and ordinary items in the future. These results suggest that scarcity goods are immediately perceived as higher in value than ordinary goods; nevertheless, scarcity is discounted more steeply than ordinary goods when delivered in the future. Despite a wealth of research focusing on temporal discounting, little attention has been paid to scarcity in the evaluation paradigm. A recent study without an evaluation task manipulated the choices between food and money delivered at different temporal periods. The behavioral data show a steeper delay discounting associated with food than with money [71]. As for electrophysiology data, the earlier the N2 component, the greater the negative deflection elicited by food, which may indicate the capture of more resources of cognitive control [71]. These N2 results may not directly support our study owing to their different paradigms; however, it strengthens the argument that our brain can perceive different types of delivered rewards at an early stage in intertemporal decision making.

## 5. Conclusions and Limitations

With behavioral experiments and field studies, burgeoning research has focused on the links between scarcity or poverty and intertemporal decisions. This study extended these notions by leveraging methods for recording brain electrophysiological activity. Our electroencephalogram data from FRN and the FAI are consistent with the well-known myopic preference for immediate satisfaction and reveal a discrepancy evaluation between scarcity items and ordinary items over time. Scarcity items are perceived as high value, but they may be devalued in the future. The results of the present study increase our understanding of the mechanisms underlying scarcity within intertemporal decision-making, which has been relatively unexamined. As for theory and practice with neuroeconomics, the current EEG findings further support the notion that scarcity manipulated by a laboratory is still evaluated with a high value, especially in the time point of now. This notion may contribute to strategies such as marketing, promotion, and rational purchasing. This conclusion, however, should be treated cautiously by practitioners; we acknowledge that these analyses had limitations. First, we did not carefully consider the responses from participants, such as the likelihood and perceived rating of scarcity items, or the discount rate. Second, in the real world, there are diverse presentations of ordinary items as well as scarcity items. The definition of scarcity has multiple meanings. It can be defined as substantially deficient or a scarcity mindset, such as scarcity of food, money, water, and time. However, it can also distinguish between resource scarcity and product scarcity [2]. The present study focused only on product scarcity, which is the scarcity of access to goods and services for purchase, which may be different from everyday life. Third, the task adopted in present study is less ecologically valid and far from real life. Similar to other neurocognitive experiments with neuroimaging or electrophysiological technique, this experiment has certain limitations in terms of task paradigm, response, and even the payment. Although the findings of the present study improve our understanding of the impact of scarcity on intertemporal decisions, much work remains to be done. Firstly, there is a need to involve more items and broaden the time scale to reflect real life. Secondly, a much more elaborate task paradigm that is accepted by more disciplines is needed. Finally, we can combine various techniques, such as functional Magnetic Resonance Imaging (fMRI), EEG, Eye-Tracking and so forth, to better explore the cognitive neural mechanism of the impact of scarcity on intertemporal decision making.

## Data Availability

The datasets analyzed in this study are available from the corresponding author on reasonable request.

## References

[1] Shah A.K., Mullainathan S., Shafir E. (2012). Some Consequences of Having Too Little. Science.

[2] Hamilton R., Thompson D., Bone S., Chaplin L.N., Griskevicius V., Goldsmith K., Hill R., John D.R., Mittal C., O’Guinn T. (2019). The effects of scarcity on consumer decision journeys. J. Acad. Mark. Sci..

[3] Jang W., Ko Y.J., Morris J.D., Chang Y.W. (2015). Scarcity Message Effects on Consumption Behavior: Limited Edition Product Considerations. Psychol. Mark..

[4] Shi X., Li F., Chumnumpan P. (2020). The use of product scarcity in marketing. Eur. J. Mark..

[5] Bassi V. (2022). Addressing social, psychological and economic barriers helps people out of extreme poverty. Nature.

[6] Huijsmans I., Ma I., Micheli L., Civai C., Stallen M., Sanfey A.G. (2019). A scarcity mindset alters neural processing underlying consumer decision making. Proc. Natl. Acad. Sci. USA.

[7] Yaple Z.A., Yu R.J. (2020). Functional and Structural Brain Correlates of Socioeconomic Status. Cereb. Cortex.

[8] Sheehy-Skeffington J. (2020). The effects of low socioeconomic status on decision-making processes. Curr. Opin. Psychol..

[9] Mani A., Mullainathan S., Shafir E., Zhao J.Y. (2013). Poverty Impedes Cognitive Function. Science.

[10] Shah A.K., Shafir E., Mullainathan S. (2015). Scarcity Frames Value. Psychol. Sci..

[11] Diesendruck G., Chiang W., Ferera M., Benozio A. (2019). Cultural Differences in the Development of a Preference for Scarce Objects. Dev. Psychol..

[12] Cook L.A., Sadeghein R. (2018). Effects of Perceived Scarcity on Financial Decision Making. J. Public Policy Mark..

[13] Zwane A.P. (2012). Implications of Scarcity. Science.

[14] Wu W., Lu H., Wu Y., Fu C. (2012). The effects of product scarcity and consumers’ need for uniqueness on purchase intention. Int. J. Consum. Stud..

[15] Sachdeva S., Zhao J.Y. (2020). Distinct impacts of financial scarcity and natural resource scarcity on sustainable choices and motivations. J. Consum. Behav..

[16] Kim J., Park J., Septianto F. (2022). The impact of socioeconomic status on preferences for sustainable luxury brands. Psychol. Mark..

[17] Liang S., Ye D., Liu Y. (2020). The Effect of Perceived Scarcity: Experiencing Scarcity Increases Risk Taking. J. Psychol..

[18] Norris A.H., Rao N., Huber-Krum S., Garver S., Chemey E., Turner A.N. (2019). Scarcity mindset in reproductive health decision making: A qualitative study from rural Malawi. Cult. Health Sex..

[19] Salerno A., Sevilla J. (2019). Scarce Foods are Perceived as Having More Calories. J. Consum. Psychol..

[20] Hilbert L.P., Noordewier M.K., van Dijk W.W. (2022). Financial scarcity increases discounting of gains and losses: Experimental evidence from a household task. J. Econ. Psychol..

[21] Adamkovič M., Martončik M. (2017). A Review of Consequences of Poverty on Economic Decision-Making: A Hypothesized Model of a Cognitive Mechanism. Front. Psychol..

[22] Haushofer J., Fehr E. (2014). On the psychology of poverty. Science.

[23] Gu D., Jiang J., Zhang Y., Sun Y., Jiang W., Du X. (2020). Concern for the future and saving the earth: When does ecological resource scarcity promote pro-environmental behavior?. J. Environ. Psychol..

[24] Stein J.S., Craft W.H., Paluch R.A., Gatchalian K.M., Greenawald M.H., Quattrin T., Mastrandrea L.D., Epstein L.H., Bickel W.K. (2020). Bleak present, bright future: II. Combined effects of episodic future thinking and scarcity on delay discounting in adults at risk for type 2 diabetes. J. Behav. Med..

[25] Omar N.A., Nazri M.A., Ali M.H., Alam S.S. (2021). The panic buying behavior of consumers during the COVID-19 pandemic: Examining the influences of uncertainty, perceptions of severity, perceptions of scarcity, and anxiety. J. Retail. Consum. Serv..

[26] Huang H., Liu S.Q., Kandampully J., Bujisic M. (2020). Consumer Responses to Scarcity Appeals in Online Booking. Ann. Tour. Res..

[27] Teubner T., Graul A. (2020). Only one room left! How scarcity cues affect booking intentions on hospitality platforms. Electron. Commer. Res. Appl..

[28] Kim S., Yoon S., Baek T.H., Kim Y., Choi Y.K. (2020). Temporal and social scarcities: Effects on ad evaluations. Int. J. Advert..

[29] Baek T.H., Yoon S. (2020). Looking forward, looking back: The impact of goal progress and time urgency on consumer responses to mobile reward apps. J. Retail. Consum. Serv..

[30] Wu Y., Xin L., Li D., Yu J., Guo J. (2021). How does scarcity promotion lead to impulse purchase in the online market? A field experiment. Inf. Manag..

[31] Bazzani A., Ravaioli S., Trieste L., Faraguna U., Turchetti G. (2020). Is EEG Suitable for Marketing Research? A Systematic Review. Front. Neurosci..

[32] Schmid P.C., Hackel L.M., Jasperse L., Amodio D.M. (2018). Frontal cortical effects on feedback processing and reinforcement learning: Relation of EEG asymmetry with the feedback-related negativity and behavior. Psychophysiology.

[33] Ohme R., Reykowska D., Wiener D., Choromanska A. (2010). Application of frontal EEG asymmetry to advertising research. J. Econ. Psychol..

[34] McInnes A.N., Sung B., Hooshmand R. (2022). A practical review of electroencephalography’s value to consumer research. Int. J. Mark. Res..

[35] Walsh M.M., Anderson J.R. (2012). Learning from experience: Event-related potential correlates of reward processing, neural adaptation, and behavioral choice. Neurosci. Biobehav. Rev..

[36] Yu M., Wu X., Huang L., Luo S. (2020). Residential mobility mindset enhances temporal discounting in the loss framework. Physiol. Behav..

[37] Chandrakumar D., Feuerriegel D., Bode S., Grech M., Keage H.A.D. (2018). Event-Related Potentials in Relation to Risk-Taking: A Systematic Review. Front. Behav. Neurosci..

[38] Sambrook T.D., Goslin J. (2015). A neural reward prediction error revealed by a meta-analysis of ERPs using great grand averages. Psychol. Bull..

[39] Ferdinand N.K., Mecklinger A., Kray J., Gehring W.J. (2012). The processing of unexpected positive response outcomes in the mediofrontal cortex. J. Neurosci..

[40] Yeung N., Sanfey A.G. (2004). Independent coding of reward magnitude and valence in the human brain. J. Neurosci..

[41] Williams C.C., Ferguson T.D., Hassall C.D., Abimbola W., Krigolson O.E. (2021). The ERP, frequency, and time–frequency correlates of feedback processing: Insights from a large sample study. Psychophysiology.

[42] Qu C., Huang Y., Wang Y., Huang Y.X. (2013). The delay effect on outcome evaluation: Results from an event-related potential study. Front. Hum. Neurosci..

[43] Burnside R., Fischer A.G., Ullsperger M. (2019). The feedback-related negativity indexes prediction error in active but not observational learning. Psychophysiology.

[44] Holroyd C.B., Yeung N. (2012). Motivation of extended behaviors by anterior cingulate cortex. Trends Cogn. Sci..

[45] Wu Y., Zhou X. (2009). The P300 and reward valence, magnitude, and expectancy in outcome evaluation. Brain Res..

[46] KreuSsel L., Hewig J., Kretschmer N., Hecht H., Coles M.G.H., Miltner W.H.R. (2012). The influence of the magnitude, probability, and valence of potential wins and losses on the amplitude of the feedback negativity. Psychophysiology.

[47] Gu R., Feng X., Broster L.S., Yuan L., Xu P., Luo Y. (2017). Valence and magnitude ambiguity in feedback processing. Brain Behav..

[48] Paul K., Vassena E., Severo M.C., Pourtois G. (2020). Dissociable effects of reward magnitude on fronto-medial theta and FRN during performance monitoring. Psychophysiology.

[49] Krugliakova E., Klucharev V., Fedele T., Gorin A., Kuznetsova A., Shestakova A. (2018). Correlation of cue-locked FRN and feedback-locked FRN in the auditory monetary incentive delay task. Exp. Brain Res..

[50] Lacey M.F., Gable P.A. (2021). Frontal Asymmetry in an approach-avoidance conflict paradigm. Psychophysiology.

[51] Deng M., Wang X., Menassa C.C. (2021). Measurement and prediction of work engagement under different indoor lighting conditions using physiological sensing. Build. Environ..

[52] Kuper N., Kaeckenmester W., Wacker J. (2019). Resting Frontal EEG Asymmetry and Personality Traits: A Meta-analysis. Eur. J. Personal..

[53] Kelley N.J., Hortensius R., Schutter D.J.L.G., Harmon-Jones E. (2017). The relationship of approach/avoidance motivation and asymmetric frontal cortical activity: A review of studies manipulating frontal asymmetry. Int. J. Psychophysiol..

[54] Cherniawsky A.S., Holroyd C.B. (2013). High temporal discounters overvalue immediate rewards rather than undervalue future rewards: An event-related brain potential study. Cogn. Affect. Behav. Neurosci..

[55] Schmidt B., Holroyd C.B., Debener S., Hewig J. (2017). I can’t wait! Neural reward signals in impulsive individuals exaggerate the difference between immediate and future rewards. Psychophysiology.

[56] Huang Y., Hu P., Li X. (2017). Undervaluing delayed rewards explains adolescents’ impulsivity in inter-temporal choice: An ERP study. Sci. Rep..

[57] Gui D., Li J., Li X., Luo Y. (2016). Temporal Dynamics of the Interaction between Reward and Time Delay during Intertemporal Choice. Front. Psychol..

[58] Blackburn M., Mason L., Hoeksma M., Zandstra E.H., El-Deredy W. (2012). Delay discounting as emotional processing: An electrophysiological study. Cogn. Emot..

[59] Zhao L., Shi Z., Zheng Q., Chu H., Xu L., Hu F. (2018). Use of Electroencephalography for the Study of Gain–Loss Asymmetry in Intertemporal Decision-Making. Front. Neurosci..

[60] Hewig J. (2018). Intentionality in frontal asymmetry research. Psychophysiology.

[61] Gu R., Lei Z., Broster L., Wu T., Jiang Y., Luo Y. (2011). Beyond valence and magnitude: A flexible evaluative coding system in the brain. Neuropsychologia.

[62] Garczarek-Bak U., Disterheft A. (2018). EEG Frontal Asymmetry Predicts Product Purchase Differently for National Brands and Private Labels. J. Neurosci. Psychol. Econ..

[63] Touchette B., Lee S.-E. (2017). Measuring Neural Responses to Apparel Product Attractiveness: An Application of Frontal Asymmetry Theory. Cloth. Text. Res. J..

[64] Gable P., Harmon-Jones E. (2008). Relative left frontal activation to appetitive stimuli: Considering the role of individual differences. Psychophysiology.

[65] Miller G., Crocker L., Spielberg J., Infantolino Z., Heller W. (2013). Issues in localization of brain function: The case of lateralized frontal cortex in cognition, emotion, and psychopathology. Front. Integr. Neurosci..

[66] Silton R.L., Heller W., Towers D.N., Engels A.S., Spielberg J.M., Edgar J.C., Sass S.M., Stewart J.L., Sutton B.P., Banich M.T. (2010). The time course of activity in dorsolateral prefrontal cortex and anterior cingulate cortex during top-down attentional control. Neuroimage.

[67] Chae H., Kim S., Lee J., Park K. (2020). Impact of product characteristics of limited edition shoes on perceived value, brand trust, and purchase intention; focused on the scarcity message frequency. J. Bus. Res..

[68] Mittone L., Savadori L. (2009). The Scarcity Bias. Appl. Psychol. Int. Rev..

[69] Ferera M., Benozio A., Diesendruck G. (2020). The Development of a Scarcity Bias. Child Dev..

[70] John M., Melis A.P., Read D., Rossano F., Tomasello M. (2018). The preference for scarcity: A developmental and comparative perspective. Psychol. Mark..

[71] Yu M., Liu T., Shi J. (2020). Food is discounted more steeply than money: Evidence from N2 and P3 responses in delay discounting tasks. Neuropsychologia.

[72] Mason L., O’Sullivan N., Blackburn M., Bentall R., El-Deredy W. (2012). I want it now! neural correlates of hypersensitivity to immediate reward in hypomania. Biol. Psychiatry.

